# CD4 T Follicular Helper and Regulatory Cell Dynamics and Function in HIV Infection

**DOI:** 10.3389/fimmu.2016.00659

**Published:** 2016-12-27

**Authors:** Brodie Miles, Shannon M. Miller, Elizabeth Connick

**Affiliations:** ^1^Division of Infectious Diseases, University of Colorado Denver, Aurora, CO, USA; ^2^Department of Immunology, University of Colorado Denver, Aurora, CO, USA; ^3^Division of Infectious Diseases, Department of Medicine, University of Arizona, Tucson, AZ, USA

**Keywords:** follicular T helper cells, follicular T regulatory cells, germinal center, broadly neutralizing antibodies, HIV

## Abstract

T follicular helper cells (T_FH_) are a specialized subset of CD4 T cells that reside in B cell follicles and promote B cell maturation into plasma cells and long-lived memory B cells. During chronic infection prior to the development of AIDS, HIV-1 (HIV) replication is largely concentrated in T_FH_. Paradoxically, T_FH_ numbers are increased in early and midstages of disease, thereby promoting HIV replication and disease progression. Despite increased T_FH_ numbers, numerous defects in humoral immunity are detected in HIV-infected individuals, including dysregulation of B cell maturation, impaired somatic hypermutation, and low quality of antibody production despite hypergammaglobulinemia. Clinically, these defects are manifested by increased vulnerability to bacterial infections and impaired vaccine responses, neither of which is fully reversed by antiretroviral therapy (ART). Deficits in T_FH_ function, including reduced HIV-specific IL-21 production and low levels of co-stimulatory receptor expression, have been linked to these immune impairments. Impairments in T_FH_ likely contribute as well to the ability of HIV to persist and evade humoral immunity, particularly the inability to develop broadly neutralizing antibodies. In addition to direct infection of T_FH_, other mechanisms that have been linked to T_FH_ deficits in HIV infection include upregulation of PD-L1 on germinal center B cells and augmented follicular regulatory T cell responses. Challenges to development of strategies to enhance T_FH_ function in HIV infection include lack of an established phenotype for memory T_FH_ as well as limited understanding of the relationship between peripheral T_FH_ and lymphoid tissue T_FH_. Interventions to augment T_FH_ function in HIV-infected individuals could enhance immune reconstitution during ART and potentially augment cure strategies.

## The Natural History and Function of T Follicular Helper Cells (T_FH_) and T Follicular Regulatory Cells (T_FR_)

T follicular helper cells were identified 16 years ago when CD4 T cells with a unique phenotype, notably abundant CXCR5 expression, were identified in the follicles and germinal centers (GCs) of secondary lymphoid tissues (1–3). T_FH_ express a unique transcriptional profile compared to extrafollicular and peripheral CD4 T cell subsets; they are a distinct population of CD4 T cells under the control of the master transcription regulator BCL-6 (4–6). T_FH_ rely on signaling through inducible T cell co-stimulator (ICOS), IL-21, IL-6, and STAT3 to develop and promote the GC response (7–9). Further, interactions with GC B cells support the development of CXCR5^hi^PD1^hi^ GC T_FH_ *via* sustained ICOS–ICOSL and CD40–CD40L binding (10). T_FH_ fail to accumulate in lymphoid tissues after immunization in the absence of B cells (11). T_FH_ provide help for maturation of B cells into plasma and memory subsets, as well as drive class switch recombination and expression of enzymes, such as activation-induced deaminase (AID) that promote somatic hypermutation (SHM) to generate highly mutated antibodies (1–3). T_FH_ are one of the main sources of IL-21, a key cytokine that promotes GC formation and maintenance, T_FH_ and B cell proliferation, SHM, and memory B cell/plasma cell differentiation (12–15). IL-21 is primarily produced by CD4 T cells and is particularly critical to generation of antigen-specific IgG antibodies and expansion of class-switched B cells and plasma cells *in vivo* [reviewed in Ref. (16)]. T_FH_ produce a variety of other cytokines including IL-4 (17), IL-17 (18), and IFNγ (19). In addition, they express increased levels of IL-10, ICOS, and CD40L compared to other T helper subsets, which allows them to positively regulate B cell differentiation and function (3, 20). Due to constraints of studying T_FH_ from lymphoid tissues, recent studies have attempted to establish a marker for T_FH_ in blood (21). While several markers have been used to define peripheral T_FH_ (pT_FH_), several groups have used CXCR5 and PD1 co-expression (22–24). In rhesus macaques receiving a modified vaccinia virus Ankara SIV vaccine, it was shown that CXCR5+ CD4 T cells accumulated in the blood at peak effector response post-immunization, and proliferating (Ki-67 +) CXCR5+ CD4 T cells in blood were directly correlated to T_FH_ and GC B cell frequency in lymphoid tissues (25). Yet, direct functional studies comparing lymphoid T_FH_ to pT_FH_ have not been done, and their relation to each other, as discussed later, remains uncertain.

More recently, T_FR_ were identified as a unique CD4 T cell subset that controls and regulates GC responses (26–28). Similar to T_FH_, T_FR_ express high levels of Bcl-6, CXCR5, ICOS, and PD-1 (26–29). T_FR_ are unique in their ability to express Blimp-1 simultaneously with Bcl-6, and express high levels of Foxp3 compared to T_FH_ (27). T_FR_ develop independently of T_FH_ from natural Treg precursors, although they rely on similar signals as T_FH_, such as CD28 and ICOS, to differentiate (27). T_FR_ are a crucial component of the GC response as they inhibit GC expansion and regulate T_FH_ and GC B cell numbers to prevent development of autoimmunity (26–28). Recent studies have shown that the function of T_FR_ and/or a skew in the balance between T_FH_ and T_FR_ frequency can lead to impaired humoral immunity (30–33). Thus, an imbalance of the T_FR_-mediated GC regulation and skewing of the GC reaction may counteract this highly regulated response and dampen the immune response to pathogens.

## T_FH_ Expand and are the Major Reservoir of HIV Replication in Chronic HIV Infection

In HIV infection prior to the development of AIDS, T_FH_ serve as the major site of virus replication (34–37). A CD4 T cell in the GC is on average 40 times more likely to be productively infected than a CD4 T cell outside of the follicle (36) and a median of 60–75% of HIV-producing cells are found within follicles in lymph nodes of untreated chronically HIV-infected individuals (35, 36). Within B cell follicles, the majority of HIV-producing cells are found in GC (38). Similarly, in chronically SIV-infected rhesus macaques without simian AIDS, virus replication is concentrated in B cell follicles in lymph nodes, spleen, and gut-associated lymphoid tissues, and these differences persist even after controlling for memory CD4 cell populations in the follicular and extrafollicular compartments (39).

Both heightened T_FH_ permissivity and factors in the follicular microenvironment play a role in promoting HIV replication within T_FH_. Tonsillar T_FH_ and GC T_FH_ are highly permissive to both X4- and R5-tropic HIV compared to other tonsillar T cell subsets *ex vivo* (38, 40). Heightened permissivity of T_FH_ is not fully explained by differences in memory subsets (as determined by CD95 expression), cellular activation (as measured by HLA-DR and CD38 expression), or chemokine HIV co-receptor expression (38). Within the microenvironment of the B cell follicle, specifically in the GC, follicular dendritic cells (FDC) bind HIV–antibody complexes *via* F_C_ and complement receptors (41). Although FDC are not productively infected, the virions bound to their surface are adjacent to T_FH_ within GCs (41–43), and these virions are highly infectious to T_FH_ (42), likely contributing to the high viral burden found in T_FH_. FDC further upregulate HIV replication in CD4 T cells through release of TNFα (40). A relative lack of cytotoxic T lymphocytes (CTL) in the follicle both in HIV (36) and SIV infection (39, 44), likely promotes replication at those sites. Most SIV-specific CTL lack a follicular homing phenotype (CXCR5+CCR7−), which may explain their failure to home to sites of virus replication in B cell follicles (39). Depletion of CD8 cells from SIV-infected macaques leads to increases in virus replication primarily in the extrafollicular zone, further supporting the notion that CTL are primarily active in the extrafollicular compartment and exert little antiviral activity within the follicle (45). Thus, T_FH_ are naturally highly susceptible to HIV, and their location within the immune privileged B cell follicle adjacent to FDC-bound virions further promotes high levels of HIV infection and replication.

Despite being highly permissive to HIV *ex vivo* and being the major virus-producing T cell subset in chronic HIV infection, the percentages of T_FH_ increase in early- and mid-stage chronic HIV (37, 46) and SIV infection (47). One of the hallmarks of HIV infection prior to AIDS is follicular hyperplasia. The follicles and GCs in HIV-seropositive lymph nodes are substantially larger in size than those in HIV-seronegative lymph nodes (48), suggesting that there are likely numerically more T_FH_ in HIV-seropositive compared to -seronegative lymph nodes in early and midstages of disease as well. Part of this expansion is likely antigen driven. In acute SIV infection, rapid formation of GC and accumulation of T_FH_, along with high p27 expression, in the follicle has been observed (49). In chronic HIV infection, virus-specific T_FH_ are expanded (46). It has been shown in mice that sustained antigenic stimulation from GC B cells is required to maintain the T_FH_ phenotype (50), further supporting the notion that antigen stimulation is key to T_FH_ expansion. It is likely that other factors besides antigen contribute to T_FH_ expansion. Cytokines known to promote T_FH_ survival, such as IL-6 (47, 51), and interferon-γ (IFN-γ) (52, 53), are increased in HIV and SIV infections, while IL-2, which inhibits T_FH_ differentiation, is decreased (52). Bcl-2, an anti-apoptotic protein, is upregulated on productively infected cells in *ex vivo* R5 infection (54). The cytokines present in the microenvironment of the T_FH_, as well as possible resistance to apoptosis, could therefore contribute to the expansion of the T_FH_ population.

## T_FH_ Functional Impairments and Their Impact on Humoral Immunity During HIV Infection

T follicular helper cells provide B cell help *via* IL-21, IL-4, CD40L, and ICOS to drive antibody production by GC B cells (55). It was shown that T_FH_ and CXCR5−PD1+ CD4 T cell populations from viremic subjects can support IgG1, IgM, and IgA production *ex vivo* (37), but numerous examples of T_FH_ deficiencies have been demonstrated in HIV infection (Table 1). B cell dysfunction has been well characterized during HIV infection, including the loss of memory B cell function, decreased numbers of GC B cells and plasma cells, hypergammaglobulinemia and spontaneous antibody production, and loss of T-dependent responses (37, 56, 57). Clinically, these deficits are manifested by increased vulnerability to bacterial infections as well as impaired responses to routine vaccinations. Increasing evidence has linked many of these deficits in humoral immunity to impaired T_FH_ function.

**Table 1:** **T follicular helper cells (T_FH_) functional impairments during HIV infection**.

T_FH_ definition	Compartment	Treatment	Impairment	Reference
CD4+CXCR5+PD-1^hi^	Lymph node	Y/N	Lower Env-specific responses than Gag-specific responses; decreased Bcl-6 expression after antiretroviral therapy (ART); increased transitional B cells; hypergammaglobulinemia	(46)

CD4+CD45RA−CXCR5+PD-1+Bcl-6+	Lymph node	Y/N	Higher Gag- and Pol-specific than Env-specific responses; harbor high levels of HIV DNA	(37)

CD4+CD45RA−CXCR5^hi^	Lymph node	N	Lower of proliferation, ICOS expression, IL-21, IL-10, and IL-4 production after PD-1 ligation	(58)

CD4+CD45RA−CCR7−CXCR5+	Spleen	N	Expansion coinciding with increased transitional B cells and lower memory B cells; disrupted transcriptional profiles in HIV-infected subjects; high levels of DNA integration	(59)

CD4+CXCR5+CCR6+CCR7+PD1+	Blood	Y/N	T_FH_ decrease in treatment-naïve subjects; increase with ART but not to healthy control levels; low IL-4 production; weak supporters of IgG production	(24)

HIV-specific, IL-21+CD4+	Blood	N	Lower breadth and magnitude of HIV-specific responses compared to IFNγ+CD4 T cells; no HIV-specific peripheral TFH responses in patients with higher viral loads	(60)

CD4+CXCR3−CXCR5+PD-1+	Blood	N	Not all CXCR5+ cells promote B cell help, only CXCR3− subsets; high T_FH_ frequency led to higher antibody neutralization scores but not decreased viral loads	(23)

CD3+CD4+CD45RA−CXCR5+CXCR3−	Blood	Y/N	Diminished B cell help during acute infection progression; increased TNFα and decreased IL-10 production that both correlate to decreased HIV-specific IgG production and increased viral load	(61)

*A summary of studies from individuals with HIV infection, including the definition of T_FH_ used, the location of T_FH_, and a brief description of their key functional impairments*.

In chronic SIV infection, a marked increase of proliferation and turnover of GC B cells was seen as T_FH_ accumulated (49). T_FH_ from lymph nodes of HIV-infected subjects did not produce IL-21 upon HIV antigen stimulation, but were able to after PMA/ionomycin stimulation (37). T_FH_ have high levels of Ki-67 expression but low rates of proliferation in uninfected tonsils (55) and HIV-infected lymph nodes (37). However, IL-21 levels have been reported as deficient in HIV-infected subjects (62), and a longitudinal study demonstrated that HIV-specific IL-21+ CD4 T cells are decreased in viremic subjects (63). In this study, only elite controllers maintained high levels of IL-21 production, and antiretroviral therapy (ART) only partially restored IL-21 levels (63). Interestingly, IL-21+ CD4 T cells from HIV-infected patients have low levels of CD40L expression (64). The loss of CD40–CD40L interactions could lead to impaired stimulation of B cells by CD4 T cells from viremic HIV-infected subjects (65). In another study, splenic T_FH_ from HIV-infected subjects demonstrated impairments in IL-4 production, along with reductions in CD40L and ICOS gene expression (59). Recently, it was demonstrated that chronically SIV-infected rhesus macaques have an expansion of Th1-biased GC T_FH_, phenotypically distinct from conventional GC T_FH_, which express CXCR3, produce high levels of IFNγ, and contain higher levels of SIV RNA (66).

## Impact of Altered T_FH_ Function on Anti-HIV Antibody Response

Deficits in T_FH_ likely contribute to the failure to develop effective antibody responses to HIV. A recent study of acute HIV seroconverters demonstrated the onset of impairments in the ability of circulating T_FH_ to stimulate HIV-specific antibody production by B cells are associated with peak viremia, suggesting that T_FH_ defects occur very early following infection (61). Most antibodies generated during infection that neutralize across clades of HIV, i.e., broadly neutralizing antibodies (bnAb), are generated after several years and show high levels of SHM resulting from extensive affinity maturation in the GC. These responses are only generated in a small fraction of infected individuals (67), and the critical components of bnAb generation are unknown (68). In both untreated and treated HIV-infected subjects, T_FH_ from lymph nodes were shown to be on average five times more sensitive to Gag than Env, with overall low T_FH_ cytokine production after Env stimulation (46). This could be due to the increased presence of Gag antigen compared to Env antigen in the lymph node of HIV-infected subjects (69) and the persistence of p24 antigen in lymph nodes after long-term ART (70). While it is not surprising there are more Gag-specific T_FH_ than Env-specific T_FH_, a lack of specificity to the HIV envelope by T_FH_ is likely one of the contributing factors to a lack of bnAb development. A loss of Gag-specific antibody response occurs during disease progression, but there is no simultaneous increase of high affinity Env-specific response (71). Thus, an early and sustained lack of Env-specific T_FH_ response could contribute to the slow development of HIV-neutralizing antibody responses and with the failure of many individuals to generate bnAbs.

While protective neutralizing antibodies and bnAbs have been structurally and genetically well characterized in HIV-infected individuals, it remains unclear how these antibodies are generated and whether or not T_FH_ can promote bnAb development. The development of bnAbs is relatively slow and shown to not strongly correlate with CD4 T cell counts, MHC II alleles, or typical patient demographics (72). However, some evidence suggests that T_FH_ function plays a role in HIV neutralization. Circulating CD4 T cells from HIV controllers and ART-treated individuals produced IL-21 when stimulated with an HIV peptide pool, but not those from HIV progressors (73). In a longitudinal assessment of acute HIV infection (12 months), treated individuals had consistently higher IL-21 production than untreated individuals, and IL-21 contributed to viral control in CD4 and CD8 T cell co-cultures *ex vivo* (73). In a cohort of chronic aviremic subjects, IL-21 production was reduced in circulating T_FH_ and supplementation of IL-21 or replacement of these subjects’ T_FH_ with T_FH_ from healthy controls led to increased production of HIV-specific antibodies by B cells *ex vivo* (74). In a cohort of HIV-infected individuals a limited proportion of patients developed bnAbs, but these patients had the highest levels of circulating, functional memory T_FH_ (23). However, their viral loads did not decline after 4 weeks, but began to decline in a few individuals at 40 weeks (23). T_FH_ frequency correlated strongly with bnAb development, thus indicating that T_FH_ are important for generating bnAb. In HIV-infected children receiving ART, circulating memory T_FH_ declined, expressed low levels of ICOS, and had a diminished capacity to produce IL-4 (75). Thus, impairments of T_FH_ function can persist in the absence of viremia. Further, in SHIV-infected rhesus macaques, the quality of T_FH_ response was correlated with the degree of SHM in virus-specific B cells and bnAb production (60). As virus-specific IL-4+ T_FH_ increased (IL-21 was not measured in this study), the amount of IgG+ virus-specific B cells and neutralizing response against HIV increased (60). Specifically, the frequency of IL-4+ and CD40L+ T_FH_ correlated strongly with the frequency of Env-specific IgG + B cells (60). This study also identified a population of IFNγ+ Env-specific T_FH_, which are less likely to provide B cell help, and these did not correlate to Env-specific IgG+ B cells. In another study, IL-21+ CD4 T cells in the periphery of HIV-infected individuals were shown to be functionally and transcriptionally equivalent to T_FH_, and Env-specific IL-21+ CD4 T cells provided higher quality B cell help than the Gag-specific subset. Env-specific IL-21+ CD4 T cells also positively correlated to protective responses of subjects who responded to vaccination in the RV144 study (76). Thus, eliciting the right type of T_FH_ help, rather than broad T_FH_ activation, is crucial to bnAb generation. Augmentation and promotion of T_FH_ function to boost this Env-specific IL-21+ CD4 T cell response could benefit future preventive vaccine trials and lead to broader specificity of anti-HIV antibodies and perhaps promote more rapid development of bnAbs in vaccinated individuals.

## T_FH_ Impairment and Their Relationship to Vaccine Responses in HIV-Infected Individuals

Individuals with chronic HIV infection typically produce poor antibody responses to immunization (77) and specifically had a high failure rate after a dose of the H1N1/09 influenza vaccine (78). In HIV seronegative individuals, the emergence of blood ICOS+CXCR5+CXCR3+ T_FH_ that are able to produce IL-21 correlated with influenza-specific B cell responses (79) and blood ICOS+IL-21+ influenza-specific T_FH_ expand after immunization and correlate to antibody responses (80). T_FH_ function in HIV-infected individuals could be important to respond to vaccinations, but research in this area is limited. In ART-treated HIV-infected individuals, responders to the H1N1/09 influenza vaccine had upregulated IL-21 production and increased IL-21 receptor expression on B cells (81). Further, B cells from HIV-infected influenza responders secreted high levels of IgG after stimulation with IL-21 and H1N1 antigen, whereas HIV-infected non-responders did not (81). Expression of AID was positively correlated to influenza neutralizing antibody responses in HIV-infected individuals, and those with the highest levels of AID expression carried protective antibodies for the longest amount of time (82).

Recently, the quality of T_FH_ responses to influenza vaccination was characterized in HIV-infected individuals. Of 16 HIV-infected subjects on ART receiving the H1N1/09 influenza vaccine, 8 subjects responded. Antibody responses were linked to the ability of pTFH to proliferate, to the ability of pT_FH_ in responders to proliferate, produce IL-21, and stimulate IgG production (22). In this study, pT_FH_ were not significantly altered in HIV-infected subjects and healthy controls at the time of vaccination, and the HIV-infected group had significantly higher frequencies of central memory pT_FH_ (22). These data indicate that although pT_FH_ were phenotypically similar in HIV-infected subjects compared to healthy controls, recall response and function of pT_FH_ is significantly impaired in HIV-infected subjects even after potent ART regimens. As B cell/pT_FH_ cocultures were performed with sorted cells it remains to be determined if T_FR_ in the periphery play a role in the dichotomy of HIV-infected responders and non-responders.

## Mechanisms That Underlie T_FH_ Dysfunction

One of the obvious causes of T_FH_ dysfunction is direct HIV infection of the T_FH_ themselves. Nevertheless, only a minority of T_FH_ are producing virus at any single time point (36), and thus this is unlikely to be the principal cause of their dysfunction. T_FH_ are characterized by high levels of PD-1 expression. Ligation of PD-1 on T_FH_ by lymph node B cells that express PD-L1, which are elevated in HIV-infected individuals, leads to decreases in IL-21 production and ICOS expression (58). Blockade of this interaction, with PD-L1 neutralizing antibodies, restores T_FH_ help to B cells and promotes IgG production (58). One report has shown that HIV infection leads to an expansion of PD-L1 expressing regulatory B cells in peripheral blood that positively correlate with increased viral load and T cell exhaustion (83), however, T_FH_ function was not examined.

Another likely cause of T_FH_ dysfunction in HIV infection is regulation by T_FR_. In mice, the magnitude of the GC reaction increased and autoimmune responses were generated when T_FR_ were unable to migrate into the follicle (84). Also in mice, it was demonstrated that excessive numbers of T_FH_ are correlated with impaired affinity maturation, and restoring a balanced ratio of T_FH_ to T_FR_ allows for generation of highly mutated, high avidity antibodies (85). Recent studies in rhesus macaques have shown that T_FR_ frequencies in secondary lymphoid tissues are increased in chronically SIV-infected animals (48, 86), while another study found decreases in T_FR_ during chronic infection (87). Reasons for discrepancies among these studies are not clear. In chronic HIV infection, T_FR_ are increased in lymph nodes (48) and spleen (59). They are also increased during acute *ex vivo* HIV infection of tonsil cells (48). In *ex vivo* HIV infection of human tonsil cells, our group found that T_FR_ inhibited ICOS expression, IL-21 production, and IL-4 production by T_FH_ (48). In another study of treatment-naïve, chronically HIV-infected subjects, the frequency of memory (CD45RA-CCR7-) T_FR_ and T_FH_ were shown to increase (59). These increases were associated with increased GC B cells; however, these cells were mostly naïve, pre-GC, and transitional B cells as opposed to memory B cells (59). Increases in T_FH_ and T_FR_ from spleen cells of HIV-infected subjects were associated with defects in the memory B cell compartment and reduced B cell help factors such as IL-4 (59). In addition, higher quality of Env-specific (gp120) antibodies in SIV-infected rhesus macaques was correlated with a lower frequency of T_FR_ (87). Neutralizing antibodies to HIV were negatively correlated to Foxp3+ Env-specific T_FH_ (T_FR_ were not excluded from T_FH_ in this work) in SHIV-infected rhesus macaques (60). Collectively, these data suggest that human T_FR_ increase during chronic HIV infection and impair T_FH_ function resulting in disruption of proper B cell differentiation and SHM. It has been shown in mouse models that the loss of T_FR_ function allows for higher levels of antibody production, but the resulting antibody is much lower affinity than if T_FR_ function is not impaired (32). Whether T_FR_ are able to control B cell responses directly, through T_FH_ impairment, or both remains to be shown.

## Memory T_FH_ in HIV-Infected Individuals

One clear area requiring more research is the development and fate of memory T_FH_ subsets. It is currently unknown if T_FH_ memory forms and is sustained inside or outside of the GC, or whether effector T_FH_ persist in chronic infections due to prolonged antigen exposure and GC maintenance (88, 89). This is especially difficult to distinguish in HIV-infected subjects, as high levels of antigens persist in the lymph nodes well after ART initiation. Effector T_FH_ are present as long as the GC persists, but if these cells become memory T_FH_ or influence the response to vaccinations in HIV-infected subjects remains to be determined. One challenge in defining memory T_FH_ and effector T_FH_ is the plasticity of phenotype of these cells. Studies in LCMV-infected mice have demonstrated that CXCR5+ memory T_FH_ downregulate PD-1, Bcl-6, IL-21, and ICOS compared to effector populations, but are able to recall effector T_FH_ phenotype upon antigen challenge, suggesting a T_FH_ lineage commitment of these memory cells (90, 91). CXCR5 has been used to distinguish memory T_FH_, but not all CXCR5+ CD4 T cells possess T_FH_ function after activation (23). Furthermore, in a mouse model, T_FH_ lost expression of Bcl-6, CXCR5, and PD-1 and acquired a memory phenotype when transferred into a mouse that did not express the cognate antigen (92). Thus, lack of a reliable phenotype for effector and memory T_FH_ populations remains a barrier to studying memory T_FH_ development and assessing memory responses (93).

Another important question is the location of the memory T_FH_ pool and whether there is crosstalk between blood and lymphatic tissues. It has been shown that circulating and lymph node-resident memory populations may develop independently and both are antigen specific with potent effector functions (94, 95). In mice, it was demonstrated that effector T_FH_ can circulate to various GCs within the same lymph node, but rarely escape to the periphery (94). Further, pT_FH_ with memory function were shown to develop independent of the GC in mice (96). As sampling pT_FH_ in the blood is more feasible than lymph node T_FH_ and circulating T_FH_ are shown to have memory and migrate to lymph nodes to stimulate B cell effector responses (95), most studies to date have focused on the function of pT_FH_ in vaccine responses of HIV-infected subjects. Highly functional pT_FH_ are reduced in viremic HIV-infected subjects, but rebound after the administration of ART (24). In this study, abundance of IgG+ memory B cells and neutralizing antibody did not strongly correlate with pT_FH_ frequency, however, pT_FH_ from HIV-infected subjects had relatively low IL-21 production in response to either SEB or Gag peptide pool stimulations and had low levels of IL-21 and IL-4 gene expression (24). This suggests that humoral responses and vaccine responses not only need to boost pT_FH_/T_FH_ numbers, but also elicit highly functional B cell help responses.

The extent to which HIV directly influences T_FH_ function, or whether HIV-driven enhancements in T_FR_ regulatory activity influences T_FH_ dysfunction, leading to poor memory and vaccine response, remains to be fully understood. In mice, circulating T_FR_ were shown to be expanded after viral infection and could potently suppress pT_FH_ function without requiring specific antigens (95). Whether T_FR_ prevent memory T_FH_ function, or prevent memory T_FH_ from reacquiring effector T_FH_ function, is an important area of future research. As T_FR_ frequency has been found to negatively correlate with bnAb generation (60, 87), it will be necessary to determine if they also disrupt the formation or activation of memory T_FH_. T_FR_ could prevent memory T_FH_ from having high quality effector responses and thus represent a barrier to generating effective vaccine responses. T_FR_ regulatory function could impair the activity of memory T_FH_ and be one of the contributing factors to failure of preventative HIV vaccinations. Further, as T_FR_ act non-specifically on target cells, they could also contribute to the relatively low efficacy of non-HIV vaccine responses in HIV-infected individuals.

## Conclusion

T follicular helper cells have a critical role in HIV immunopathogenesis. They proportionately expand compared to total CD4 T cells during chronic disease and are the major virus-producing cells during asymptomatic disease, thereby driving disease progression. They also exhibit multiple functional deficits that impair development of robust humoral immunity to pathogens, including HIV itself. Mechanisms underlying T_FH_ impairment likely include direct infection of T_FH_, suppressive factors in the GC milieu such as PD-L1 expression on B cells, and T_FR_. Strategies to augment T_FH_ immunity remain to be developed, but potential interventions include administration of IL-21 as well as inhibition of T_FR_ responses. Such strategies need to be developed cautiously as unintended consequences of these interventions, such as development of autoimmunity due to excessive inhibition of T_FR_, could be deleterious. A better understanding of the nature of memory T_FH_ populations is also essential in order to develop and test interventions. Knowledge of factors that influence T_FH_ function in HIV infection could lead to improved immune reconstitution in ART-treated individuals and potentially augment strategies to cure HIV infection.

## Author Contributions

All the authors listed have made substantial, direct, and intellectual contribution to the work and approved it for publication.

## Conflict of Interest Statement

The authors claim no financial conflicts of interest, and no outside parties influenced the writing of this manuscript.
